# A Self-Organizing Fuzzy Logic Classifier for Benchmarking Robot-Aided Blasting of Ship Hulls

**DOI:** 10.3390/s20113215

**Published:** 2020-06-05

**Authors:** M. A. Viraj J. Muthugala, Anh Vu Le, Eduardo Sanchez Cruz, Mohan Rajesh Elara, Prabakaran Veerajagadheswar, Madhu Kumar

**Affiliations:** 1 Engineering Product Development Pillar, Singapore University of Technology and Design, 8 Somapah Rd, Singapore 487372, Singapore; eduardo_sanchez@sutd.edu.sg (E.S.C.); rajeshelara@sutd.edu.sg (M.R.E.); prabakaran@sutd.edu.sg (P.V.); 2 Optoelectronics Research Group, Faculty of Electrical and Electronics Engineering, Ton Duc Thang University, Ho Chi Minh City 700000, Vietnam; leanhvu@tdtu.edu.vn; 3 Brightsun Marine Pte Ltd, 9 Tuas Ave 8, Singapore 639224, Singapore; madhu@oceaniarobotics.com

**Keywords:** self-organizing fuzzy logic classifier, benchmarking blasting quality, hydro blasting, ship hull maintenance, robotics for ship maintenance industry

## Abstract

Regular dry dock maintenance work on ship hulls is essential for maintaining the efficiency and sustainability of the shipping industry. Hydro blasting is one of the major processes of dry dock maintenance work, where human labor is extensively used. The conventional methods of maintenance work suffer from many shortcomings, and hence robotized solutions have been developed. This paper proposes a novel robotic system that can synthesize a benchmarking map for a previously blasted ship hull. A Self-Organizing Fuzzy logic (SOF) classifier has been developed to benchmark the blasting quality of a ship hull similar to blasting quality categorization done by human experts. Hornbill, a multipurpose inspection and maintenance robot intended for hydro blasting, benchmarking, and painting, has been developed by integrating the proposed SOF classifier. Moreover, an integrated system solution has been developed to improve dry dock maintenance of ship hulls. The proposed SOF classifier can achieve a mean accuracy of 0.9942 with an execution time of 8.42 µs. Realtime experimenting with the proposed robotic system has been conducted on a ship hull. This experiment confirms the ability of the proposed robotic system in synthesizing a benchmarking map that reveals the benchmarking quality of different areas of a previously blasted ship hull. This sort of a benchmarking map would be useful for ensuring the blasting quality as well as performing efficient spot wise reblasting before the painting. Therefore, the proposed robotic system could be utilized for improving the efficiency and quality of hydro blasting work on the ship hull maintenance industry.

## 1. Introduction

Routine dry dock maintenance on the outer hull of ships is essential for the efficient and sustainable operation of shipping [1,2]. Improper maintenance of ship hulls may lead to an increase of the fuel consumption due to the surface roughness [2,3]. In addition to that, safety issues can also be aroused due to the improper hull maintenance. Typically, maintenance work on ship hulls needs to be carried out every 4–5 years [4]. One of the major maintenance work on ship hull is the blasting work carried out for removing rust and adherence [2]. Hydro blasting or abrasive blasting is commonly used for this purpose. In most of the typical cases, these blasting works are carried out by human workers with the aid of semi-automatic devices [2]. Furthermore, inspections of hull surfaces are done by humans to identify the areas that need to be blasted as well as to evaluate the quality of a blasting work [2]. Thereby, the conventional ship hull maintenance work suffers from concerns in accuracy, efficiency, cost, and safety.

Many robotic solutions have been emerged for resolving the issues associated with conventional maintenance work that requires extensive human labor in diverse domains [5,6,7,8]. Similarly, many robots and devices have been developed for inspection and maintenance work of ship hulls to resolve the shortcoming of the conventional methods discussed above [9]. In this regard, many ground-based robots and robotic systems have been introduced with the intention of automating dry dock maintenance work of ships hulls [10,11,12,13]. These robots require the support of external structures such as rail mechanisms, gantry cranes, and cherry pickers for facilitating the movements along a ship hull. A system that uses a set of stationary lidars and cameras for detecting corrosion spots in a ship hull has been proposed in [14]. However, these ground robots have limited access to ship hulls due to hindrances caused by geometric constraints and occultations. Furthermore, it is required to deploy either a set of systems or facilitates the movement of the structures around a ship for covering an entire ship hull. Possibility of using Unmanned Area Vehicles (UAV) such as quadcopters and hexacopters for inspection of ship hulls has also been investigated since UAVs have proven the ability to inspect confined and limited access areas in other application domains [9,15]. Nevertheless, the usability of UAVs is limited in inspection of ship hulls due to the lack of operation for a longer time and distance as well as low accuracies in positioning.

Robots that can climb on ship hulls are preferred for inspection and maintenance work of ship hulls due to their performance benefits [16,17]. A climbing robot based on magnetic adhesion has been developed for inspecting long welding lines on ship hulls [18]. MIRA [19] is another climbing robot developed for the inspection of ship hulls. The wheels of this robot are integrated with permanent magnets fixed on a flexible strip to provide the climbing ability. A mobile robot with a novel design of magnetic wheels based on Halbach array has been introduced in [20] for dry dock inspections. A tracked mobile robot that is capable of adapting to the uneven surfaces has been developed for ship hull inspection [21]. The self-adaptability of the robot allows it to move through uneven surfaces. Since magnetic wheels are often used for inspection robots intended for ship hulls, in depth analysis on magnetic flux distribution of wheels for climbing robot has also been conducted [22]. Furthermore, optimization of a magnetic wheel for a grit blasting robot has been investigated [23]. Apart from the climbing robot with magnetic adhesion, robots based on negative pressure adhesion have also been developed [17,24]. The work [24] analyzed the variation of required negative pressure in different conditions such as in wet conditions. Nevertheless, the scope of the work cited above is limited to the design and development of climbing robots or their adhesion mechanisms for inspection of ship hulls; the methods for automatic inspection and maintenance such as vision-based corrosion detection, are not discussed within the scope of the work.

A magnetic crawler robot with a monocular-camera based vision processing method for inspecting ship hulls has been developed [25]. The vision processing method is capable of constructing a mosaic image, which can produce a metric representation of the areas inspected by the robot, from the multiple images captured from different positions of the robot. Many computer-vision based methods for detecting corrosion in metal structures, including ship hulls and steel bridges, have been developed [26]. According to the outcomes of the survey [26], the color is the principal representative feature for identifying the corroded areas. Furthermore, the cited survey concludes that learning-based methods could perform better than the non-learning methods in detecting corrosion through vision. A method for a surface corrosion grade classification of metals has been proposed using a deep convolution neural network [27]. However, the method was developed to detect the correction grade based on microscopic images of the surface. A microscopic image could cover only a minimal area of the environment to be inspected due to the constraints of magnification. If microscopic images were taken to cover a ship hull, a considerably large number of images would need to be taken for covering the whole surface. The vision system has to be directed toward very near proximity areas many times in this regard. Therefore, the inspection process would be prolonged, hindering the feasibility of adopting the method proposed in [27] for a robot intended for inspecting the corrosion grade of ship hulls. A vision-based defect detection method based on statistical approaches built upon circular histograms entropy analysis has been introduced to identify the rusted regions of ship hulls [28]. Nevertheless, the proposed method has been verified merely using offline images and have not been validated in the real-world application context.

The ability to detect corrode areas of ship hulls from a set of stationary cameras by using histogram-based background detection and adaptive thresholding methods have been investigated in [14]. According to the outcomes, these methods are effective only when the inspected zones have a majority of non-corroded areas. The work [29] proposed an aerial robot equipped with a feedforward neural network trained for corrosion detection of ship hulls. The proposed neural network is capable of detecting the areas of corrosions/coating breakdowns of ship hulls. An automated visual inspection method that can detect defected areas for automated spot blasting has been proposed in [30]. The cited work proposes a wavelet transformation combined with an entropy-based method for detecting the corrode spots. The proposed method has been implemented on a crane-based ground robotic system and verified the abilities.

Nevertheless, all the vision-based methods discussed above are intended for detecting corroded areas on a surface. The blasting quality needs to be classified into a few categories for benchmarking a hydro blasting work. Furthermore, the surface appearance becomes different after a blasting process. Hence, the methods discussed above could not be adopted for benchmarking the performance of a hydro blasting process. In addition to that, much of the work is limited to the development of vision-based detection mechanisms for robots, and the ways for utilizing the detection outcomes as a fully integrated system for synthesizing a benchmarking map for an already blasted ship hull are not considered within the scopes. Therefore, this paper proposes a novel vision-based benchmarking method for hydro blasting. The proposed benchmarking method can classify the hydro blasting quality into three categories, good, medium, and bad. A Self-Organizing Fuzzy logic (SOF) classifier is proposed to realize the classification into the benchmarking categories. In addition to that, a hydro blasting robotic system consists of a hydro blasting robot and a benchmarking robot has been designed and developed. An overview of the proposed robotic system is given in Section 2. Section 3 presents the theoretical backgrounds of the proposed SOF classifier. Particulars on experimental validation are discussed in Section 4. Concluding remarks are given in Section 5.

## 2. System Overview

### 2.1. Context of Application

**Figure 1:**
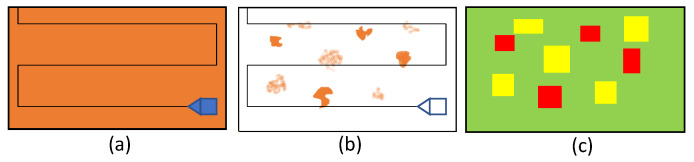
The overall sequence of the application context of the proposed robotic system. (**a**) Blasting robot is sent in a zig-zag path for uniformly blasting the area; (**b**) The benchmarking robot is sent in the previously blasted area to benchmark the blasting quality; (**c**) The benchmarking map that represents the quality of blasting in different areas.

**Figure 2:**
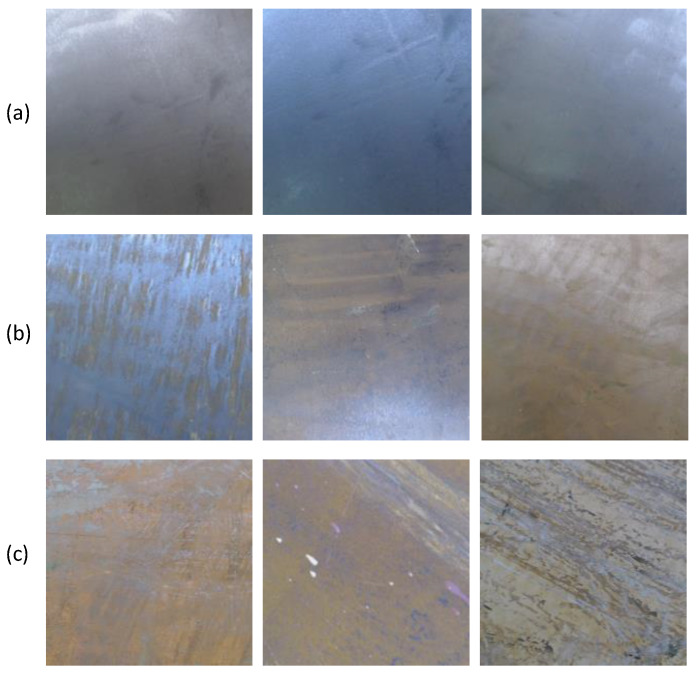
Categorization of the blasting quality considered for benchmarking. (**a**) Appearances of good areas; (**b**) Appearances of medium quality areas; (**c**) Appearances of bad quality areas.

The context of the application of the proposed robotic system is explained with the aid of Figure 1. The application context is to remove the layer of rust in a ship hull through hydro blasting for applying a new painting. Initially, a robot equipped with hydro blasting capability is sent on the ship hull in a zig-zag path to cover the entire area to be blasted (as shown in Figure 1a). While navigating in the given path, the robot is expected to continuously and uniformly perform the hydro blasting in the area. Typically, a hydro blasting robot is capable of removing the rust layer to a greater extent. Nevertheless, the rust removal would not always be uniform throughout a given area, and there would be partially removed areas or completely unremoved areas. Thus, the blasting quality is usually inspected by human experts after performing a blasting cycle. ISO 8501-1 standard is used as a guideline in this regard. A blasted surface with either SA 2.5 or SA 3 standard quality (according to ISO 8501-1) is considered as “good” in the work presented in the paper, where the blasting quality is adequate for processing with painting. Typical appearance of a ship after a good blasting is shown in Figure 2a. According to the standard, a blasted surface free from visible oil, grease, and dirt and from mill scale, rust, paint coatings, and foreign matters, when the surface is inspected without magnification, can be considered as at least SA 2.5 quality. Slight traces of contamination in the form of spots or stripes are allowed in SA 2.5 while the surface should have uniform metallic color without any traces to be considered as SA 3 quality. Areas with standard blasting quality SA 1 and SA 2, where poorly adhering rust, paint coatings, and foreign matters could be observed without magnification, are considered as medium quality in the work presented in this paper. Appearances of areas with medium blasting quality are given in Figure 2b. The medium quality areas are expected to have a light reblasting on it. If a surface blasting quality is below the quality definition of SA 2 (e.g., an area with a completely unremoved coat), it is considered bad quality blasting. The areas identified as bad quality blasting are expected to have a full reblasting on them. Examples for areas categorized as bad quality blasting is given in Figure 2c. If the new painting was applied on top of a ship hull with existing rust particles, then it would not last for a long time. Thereby, it is necessary to ensure that a ship hull is completely blasted before applying the paint.

To ensure that a ship hull is uniformly blasted in good condition, a second robot is sent on the ship hull in a zig-zag path as shown in Figure 1b, to benchmark the work done by the blasting robot. After completion of the inspection by the robot, a benchmarking map for the hull area is developed as shown in Figure 1c, where it indicates the quality of blasting in different segments of areas. The areas shown in green represent the areas with good quality blasting (areas where the appearance is similar to Figure 2a). The areas given in yellow represent the areas where the quality of blasting is medium (areas where the appearance is similar to Figure 2b). The areas where the blasting quality is bad (areas where the appearance is similar to Figure 2c) is represented in red. After generating the benchmarking map of the hull area, it is expected to resend the blasting robot to perform selective blasting on medium and bad quality areas. This benchmarking map would be useful in improving the efficiency of the robot by planning an efficient navigation plan for the blasting robot and performing selective blasting with different blasting parameters (for medium areas low pressure, for bad areas high pressure, etc.). Therefore, the robotic system proposed in this paper would be highly beneficial in improving the automated ship hull blasting and inspection work.

### 2.2. Functional Overview

**Figure 3:**
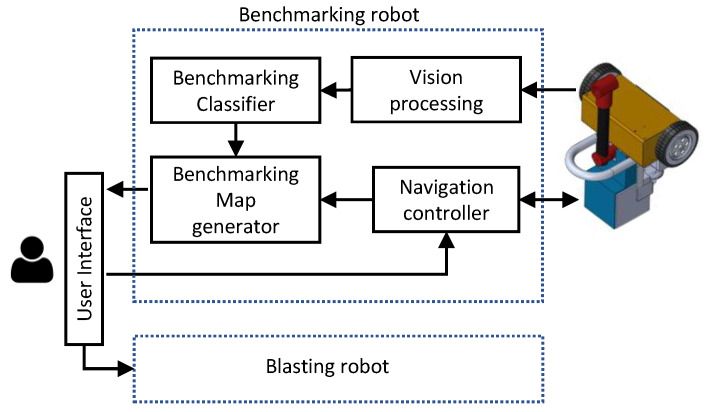
Functional overview.

The functional overview of the proposed robotic system is depicted in Figure 3. An operator can control the navigation path of the benchmarking robot through the user interface. The navigation controller of the benchmarking robot is responsible for performing the low-level functionalities related to navigation in a given trajectory such as localization and locomotion motor controlling. For localization, the robot uses a beacon-based off the shelf localization system. The vision feedback from the camera attached to the robot is processed by the vision processing module to extract the features.

**Figure 4:**
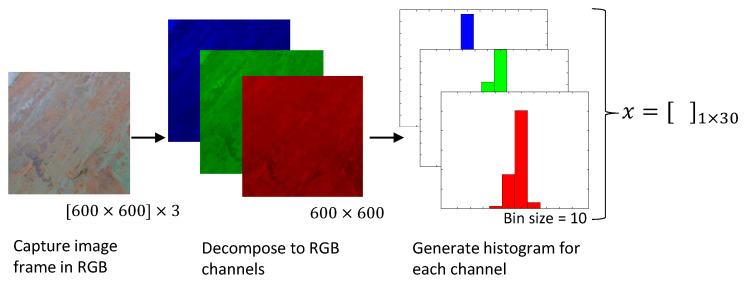
Steps of image processing for feature extraction.

The main steps within the vision processing module are explained in Figure 4. The vision processing module captures image frames from the incoming vision feed. This capturing is done in 600 × 600 size. Then, the image information of each frame is separated into R, G, and B components. Then, for each color component, a histogram is generated. The bin size of a histogram is configured to 10. Hence, there are 10 parameters for each color component yielding to 30 parameters as the output of the vision processing module (for altogether for R, G, and B components 30 parameters). This data set is denoted as *x*, and it is a row vector with 30 elements. The output of the vision processing module, *x* is fed to the benchmarking classifier for each captured frame.

The benchmarking classifier labeled each image frame to either of the considered benchmarking categories, good, medium, and bad. The benchmarking classifier has been developed using a Self-Organizing Fuzzy logic (SOF) classifier (A detailled description is given in Section 3). The classification results of the incoming image frames are then sent to the benchmarking map generator. The corresponding location of a captured image is retrieved from the navigation controller. Then the corresponding location is tagged with the classified results to generate the benchmarking map. The created benchmarking map could be accessible through the user interface. In addition to that, the generated benchmark map could be transferred to the blasting robot through the user interface for reblasting the areas identified as medium and bad.

### 2.3. Robot Platform

**Figure 5:**
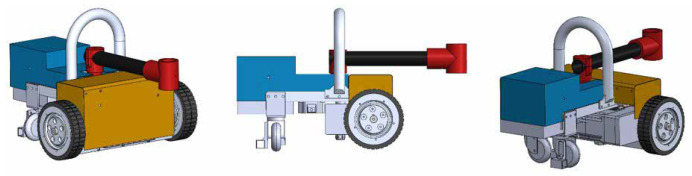
Hornbill Design.

Hornbill is a differential drive robot designed for ship hull maintenance. The robot uses magnets as principal support to adhere and navigate across the metallic surface of the vessel, besides it incorporates custom design wheels for water displacement to maximize the traction in the presence of water. The robot’s architecture incorporates a multipurpose arm that can be used for hydro blasting, painting, and surface benchmarking. Figure 5 depicts the robot’s design.

**Figure 6:**
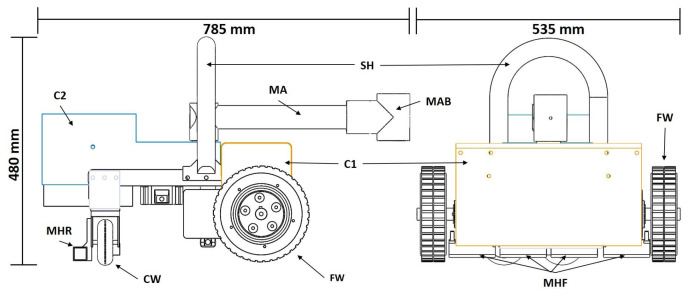
Part Diagram.

A complete part diagram of the robot is shown in Figure 6 for reference. The robot dimensions are 535 × 785 × 480 mm including the multipurpose arm. At the front axis, it has 2 DC motors coupled to a gear head that transmits 55 Nm of torque each to the surface by using 2 rubber wheels of 200 mm of diameter (FW). The robot’s frame is made of Aluminium 6061 Alloy to minimize any possible hazard to occur due to the presence of high magnetic forces. The frame also has a safety handler (SH) to easy its deployment on a ship’s hull. The robot’s design also includes a waterproof cover (C1, C2) to isolate the electric components from the liquids.

The robot is designed to carry 300 kg of payload including its weight, for this purpose the robot includes 10 grade N50 Neodymium squared magnets of 200 × 25 × 25 mm, 8 magnets are located at the front axis using 4 magnet holder (MHF) and 2 at the rear (MHR) right next to 2 castor wheels (CW). The magnets are placed at 5 mm from the surface, and they provide a combined pull force of 300 kg.

**Figure 7:**
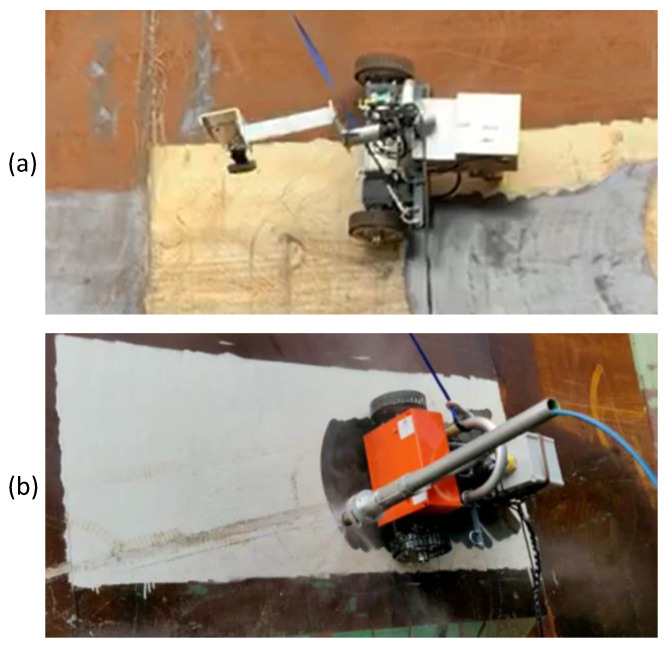
(**a**) Hornbbill is used as the bechmarking robot; (**b**) Hornbill is performing hydro blasting.

Hornbill has been designed to be reconfigurable in terms of the task that needs to perform. Therefore, the robot has attached a multipurpose arm (MA) made of a hollow aluminum tube, at one end it is coupled a DC motor of 25 Nm of torque that allows the arm to swing, and at the other end it has a bracket (MAB) that can be used to link diverse tools depending on what is the robot needed to perform between hydro blasting, painting, and surface benchmarking. To perform surface benchmarking, a camera has to be attached to the arm bracket (MAB) oriented parallel to the hull surface. Situations, where Hornbill is used as a benchmarking robot and a hydro blasting robot, are shown in Figure 7.

A set of beacons have to be placed along the section of the ship’s hull where the robot operates. The communication of a beacon placed on the robot with the surface beacons is used to determine the robot’s absolute position and orientation. In addition to the information from the beacons, the information from wheel encoders and inertia measurement unit is fused together to improve the accuracy of the localization within the workspace. This localization facilitates the autonomous navigation of the robot in a given trajectory.

## 3. Self-Organizing Fuzzy Logic (SOF) Classifier

A Self-Organizing Fuzzy logic (SOF) classifier [31] is proposed for the benchmarking classifier. The architecture of the SOF classifier was originally proposed by Gu and Angelov [31] in 2018. The underlying theoretical rationales and mathematical proving of the architecture, such as analysis of convergence, have been analyzed in the cited work. Furthermore, the performance of the SOF classifier has been compared against the other method using well-known offline testing data such as data set for optical character recognition. Nevertheless, a SOF classifier has not been proposed for benchmarking the quality of hydro blasting. A SOF classifier was selected for this application based on the following reasons.

- The benchmarking statuses are defined based on human expert knowledge, and the benchmarking categorization is performed based on the three fuzzy linguistic descriptors, good, medium, and bad. Moreover, the benchmarking classifier should emulate the human expert knowledge in the classification process. Fuzzy logic has been proven to be well suited for replicating the human expert knowledge that can be represented through linguistic expressions [32,33,34]. Furthermore, fuzzy logic has the ability to cope with imprecise sensor information [35,36,37]. Therefore, a method based on fuzzy concepts would be expected to perform well in this specific application.
- A human interpretable and explainable set of rules is generated after the training of a SOF classifier. Explainable intelligent techniques are preferred for ensuring transparency and trust of safety in this sort of industrial application, which might become hazardous from undesired control actions that might be performed by a robot [38]. Furthermore, the set of rules can be tailored based on expert knowledge.
- A SOF classifier is a highly efficient model with high classification accuracy [31,39]. Therefore, it requires lower computational power with respect to the other existing models. In addition to that, a SOF classifier does not require dedicated optimized hardware such as GPU cores for the computation. Moreover, a SOF model is comparatively lightweight.
- Many existing classification models rely heavily on prior assumptions on data generation models and user-defined trial and error parameters such as learning rate and the size of the network. In most of the practical cases, the assumptions on data generation are often too tough to be sustained, and user-defined parameters are often troublesome to define due to the insufficient prior knowledge of the problem. In contrast, a SOF classifier is nonparametric, and it does not require an assumption on data generation models and parameter knowledge about the problem of interest [31].

**Figure 8:**
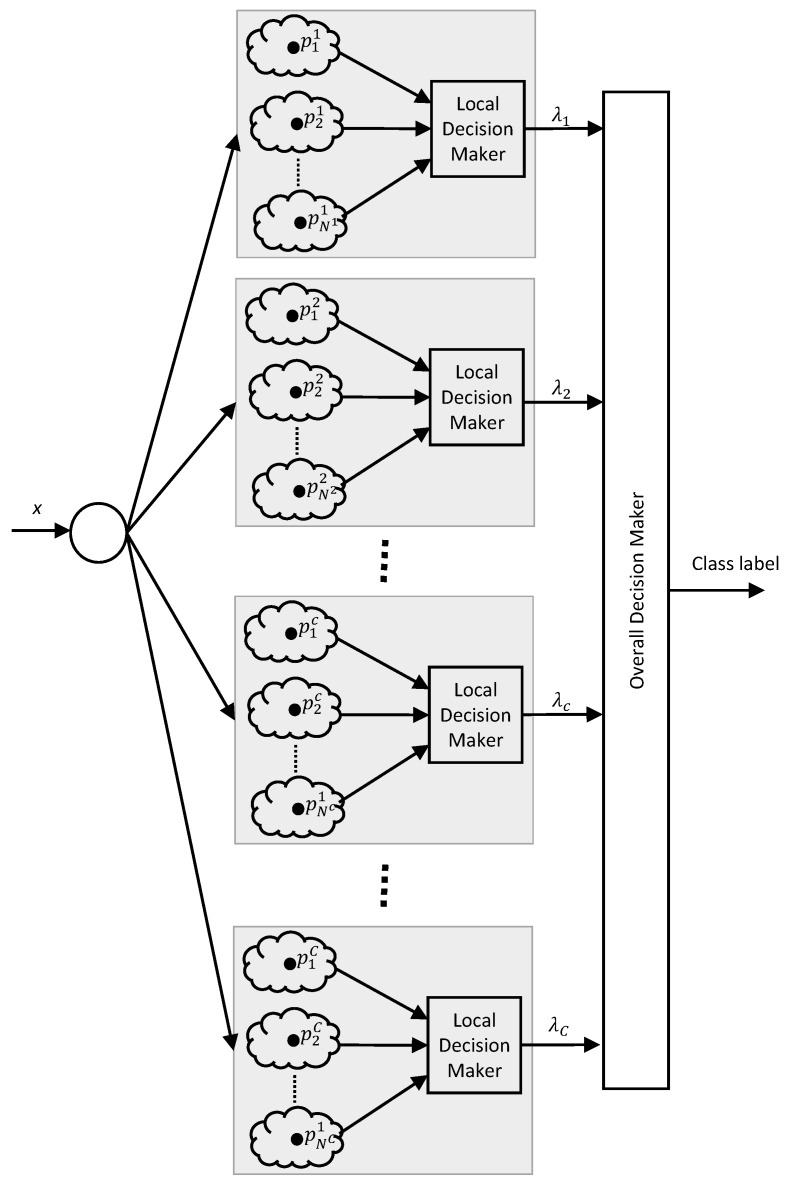
Architecture of Self-Organizing Fuzzy Logic (SOF) classifier.

The architecture of the SOF classifier is depicted in Figure 8. It is designed to assign a class label to a given data sample, *x* based on trained knowledge. It should be noted that *x* can be a row vector with any dimension. The trained knowledge is stored as a set of zeroth-order AnYa type fuzzy rules [40]. An AnYa type fuzzy rule has the form given in (1). The antecedent of an AnYa type fuzzy rule is in a nonparametric vector form, and it does not require membership functions. Here, the symbol, ∼ denotes the similarity between a data sample and a data cloud. This similarity is similar to the concept of degree of membership in the Mamdani type or Sugeno type fuzzy inference system. ${\left\{p\right\}}^{c}=\left\{p_{1}^{c},p_{2}^{c},\ldots ,p_{N^{c}}^{c}\right\}$ is the set of prototypes belonging to $c^{\mathrm{th}}$ class, and $N^{c}$ is the number of prototype in ${\left\{p\right\}}^{c}$. These prototypes are the centers of the data clouds. The data clouds and the corresponding fuzzy rules are formed during the training phase of the SOF classifier.

(1) $$\mathrm{IF}\;\left(x∼p_{1}^{c}\right)\mathrm{OR}\left(x∼p_{2}^{c}\right)\mathrm{OR}\ldots \mathrm{OR}\left(x∼p_{N^{c}}^{c}\right)\;\mathrm{THEN}\;\left(class\;c\right)$$

To identify the class corresponding to an input data sample (i.e., *x*), the firing strength of $c^{\mathrm{th}}$ fuzzy rule, $\lambda^{c}\left(x\right)$ is evaluated as in (2) by the local decision-maker where d(x,p) denotes distance/dissimilarity between the two data points. Common distance/dissimilarity measures such as Euclidean, Mahalanobis, and Cosine distance can be used in this purpose. Then, the class label of the data sample is assigned by the overall decision-maker using the winner-takes-all method as in (3). For c=1,2,…,C.

(2) $$\lambda^{c}\left(x\right)=\max_{p\in {\left\{p\right\}}^{c}}\left(e^{-d^{2}\left(x,p\right)}\right)$$

(3) $$label=\underset{c=1,2,\ldots ,C}{arg max}\left(\lambda^{c}\left(x\right)\right)$$

The classifier identifies the prototypes from each class and forms the data clouds. A zeroth-order AnYa type fuzzy rule for each class is then formulated. The training process is independent for different classes, and there is no influence from the training of one class to another class. Thereby, the training process can be explained based on $c^{\mathrm{th}}$ class such that c=1,2,…,C. Suppose data sample set of $c^{\mathrm{th}}$ class is denoted by ${\left\{x\right\}}_{K^{c}}^{c}=\left\{x_{1}^{c},x_{2}^{c},\ldots ,x_{K^{c}}^{c}\right\}$ such that ${\left\{x\right\}}_{K^{c}}^{c}\subset {\left\{x\right\}}_{K}$, where $K^{c}$ is the number of data samples belonging to $c^{\mathrm{th}}$ class and *K* is the total number of data samples in the dataset. All the data in a training data sample may not be unique and the same data sample may be repeated. The corresponding unique data sample set and their frequency of appearance are considered as ${\left\{u\right\}}_{U_{K}^{c}}^{c}=\left\{u_{1}^{c},u_{2}^{c},\ldots ,u_{U_{K}^{c}}^{c}\right\}$ and ${\left\{f\right\}}_{f_{K}^{c}}^{c}=\left\{f_{1}^{c},f_{2}^{c},\ldots ,f_{U_{K}^{c}}^{c}\right\}$ respectively, where $U_{K}^{c}$ is the number of unique data sample belonging to $c^{\mathrm{th}}$ class, and $U_{K}$ is the total number of unique samples in the dataset. Moreover, this definitions lead to $\sum_{c=1}^{C}K^{c}=K$ and $\sum_{c=1}^{C}U_{K}^{c}=U_{K}$. The main steps of the training of SOF classifier are given below.

**Step 1:** The multimodal density [41] of $i^{\mathrm{th}}$ unique data sample of $c^{\mathrm{th}}$ class, $D_{K^{c}}^{MM}\left(u_{i}^{c}\right)$ is calculated as in (4) where $i=1,2,\ldots ,U_{K}^{c}$ and $d(x_{i},x_{j})$ denotes distance/dissimilarity between the two data points.
  (4) $$D_{K^{c}}^{MM}\left(u_{i}^{c}\right)=f_{i}^{c}\frac{\sum_{l=1}^{K^{c}}\sum_{j=1}^{K^{c}}d^{2}\left(x_{l}^{c},x_{j}^{c}\right)}{2K^{c}\sum_{j=1}^{K^{c}}d^{2}\left(u_{i}^{c},x_{j}^{c}\right)}$$
**Step 2:** The sample ${\left\{u\right\}}_{U_{K}^{c}}^{c}$ is sorted according to the multimodal density and mutual distances calculated in step 1. The sorted sample set is denoted by $\left\{r\right\}=\left\{r_{1},r_{2},\ldots ,r_{U_{K}^{c}}\right\}$, where $r_{1}$ is given in (5) and the rest are obtained as in (6). It should be notated that $u_{i}^{c}$ corresponding to $r_{k}$ is excluded in each run of (6), and the process is repeated for all the data in the sample.
  (5) $$r_{1}=\underset{i=1,2,\ldots ,U_{K}^{c}}{arg max}\left(D_{K^{c}}^{MM}\left(u_{i}^{c}\right)\right)$$
  (6) $$r_{j}=\underset{u_{i}^{c}\in {\left\{u\right\}}_{U_{K}^{c}}^{c}}{arg min}\left(d\left(r_{k},u_{i}^{c}\right)\right)\;\;\;\mathrm{for}\;j=2,3,\ldots ,U_{K}^{c}$$
**Step 3:** The multimodal density set after the sorting in step 2 is taken as $\left\{D_{K^{c}}^{MM}\left(r\right)\right\}$. The initial set of prototypes, ${\left\{p\right\}}_{0}$ is generated by considering the condition given in (7). Moreover, the local maxima of $\left\{D_{K^{c}}^{MM}\left(r\right)\right\}$ are taken for ${\left\{p\right\}}_{0}$.
  (7) $$\mathrm{IF}\;(D_{K^{c}}^{MM}\left(r_{i}\right)>D_{K^{c}}^{MM}\left(r_{i+1}\right)\;\mathrm{AND}\;(D_{K^{c}}^{MM}\left(r_{i}\right)>D_{K^{c}}^{MM}\left(r_{i-1}\right)\;\;\;\mathrm{THEN}\;\;\;\left(r_{i}\in {\left\{p\right\}}_{0}\right)$$
**Step 4:** The nearby data samples are attracted to form data clouds resembling Voronoi tesselation [42]. The assignment of a data sample to a cloud is determined based on the winning prototype obtained as in (8).
  (8) $$\mathrm{Winning}\;\mathrm{Prototype}=\underset{p\in \left\{p_{0}\right\},x_{i}\in {\left\{x\right\}}_{K^{c}}^{c}}{arg min}\left(d\left(x_{i},p\right)\right)$$
**Step 5:** The set of centers of the formed data clouds, $\left\{\varphi_{0}\right\}$ are identified. $\left\{\varphi_{0}\right\}$ is equivalent to $\left\{p_{0}\right\}$. The multimodal density at the center of $i^{\mathrm{th}}$ cloud is calculated as in (9) where $S_{i}$ is the number of members in $i^{\mathrm{th}}$ cloud, and *n* is the number of clouds formed.
  (9) $$D_{K^{c}}^{MM}\left(\varphi_{i}\right)=S_{i}\frac{\sum_{l=1}^{n}\sum_{j=1}^{n}d^{2}\left(\varphi_{l},\varphi_{j}\right)}{2n\sum_{j=1}^{n}d^{2}\left(\varphi_{i},\varphi_{j}\right)}$$
**Step 6:** The set of centers of neighboring data clouds of $i^{\mathrm{th}}$ data cloud, ${\left\{\varphi \right\}}_{i}^{neighbor}$ is identified for each *i* based on the condition given in (10) such that $\varphi_{j}\in {\left\{\varphi \right\}}_{0}$ and $\varphi_{j}\neq \varphi_{i}$. Here, $G_{K^{c}}^{c,L}$ defines the average radius of local influential area around each data sample. This parameter is calculated iteratively as given in (11) based on the granularity level $L\in Z^{+}$ defined by user. Here, $Q_{K^{c}}^{c,L}$ is the number of pairs of data samples where the distance between a pair is less than $G_{K^{c}}^{c,L}$ for L≠=1. When L=1, $Q_{K^{c}}^{c,L}$ is the number of pairs of data samples where the distance between a pair is less than the average distance, ${\bar{d}}_{K^{c}}^{c}$.
  (10) $$\mathrm{IF}\;(d^{2}\left(\varphi_{i},\varphi_{j}\right)\leq G_{K^{c}}^{c,L}\;\mathrm{THEN}\;\left(\varphi_{i}\in {\left\{\varphi \right\}}_{i}^{neighbor}\right)$$
  (11) $$G_{K^{c}}^{c,L}=\left\{\begin{array}{cc} \frac{\sum_{x,y\in {\left\{x\right\}}_{K^{C}}^{c},x\neq y,d^{2}\left(x,y\right)\leq {\bar{d}}_{K^{c}}^{c}}d^{2}\left(x,y\right)}{Q_{K^{c}}^{c,L}} & \mathrm{if}\;L=1\\ \frac{\sum_{x,y\in {\left\{x\right\}}_{K^{C}}^{c},x\neq y,d^{2}\left(x,y\right)\leq G_{K^{c}}^{c,L-1}}d^{2}\left(x,y\right)}{Q_{K^{c}}^{c,L}} & \mathrm{otherwise} \end{array}\right.$$
**Step 7:** The set of representative prototypes of $c^{\mathrm{th}}$ class, ${\left\{p\right\}}^{c}$ are identified by evaluating the condition given in (12).
  (12) $$\mathrm{IF}\;\left(D_{K^{c}}^{MM}\left(\varphi_{i}\right)>\max_{\varphi \in {\left\{\varphi \right\}}_{i}^{neighbor}}\left(D_{K^{c}}^{MM}\left(\varphi \right)\right)\right)\;\mathrm{THEN}\;\left(\varphi_{i}\in {\left\{p\right\}}^{c}\right)$$
**Step 8:** A zeroth order AnYa type fuzzy rule is created for $c^{\mathrm{th}}$ class in the format given in (1), where $N^{c}$ is the number of representative prototypes.

## 4. Results and Discussion

### 4.1. Data Collection and Training, and Classification Performance

The data set required for the training and testing was prepared by capturing images of blasted ship hulls through the robot’s camera. The captured images were manually labeled to benchmarking categories with support of expert knowledge. For each benchmarking class, 1850 images were gathered, yielding the total size of the data set to 5550 images. Geometrical transformations, such as rotation, were applied to the data set to prevent the possible overfitting. The data set was randomly divided into two subsets for training and testing in the ratio of 80:20.

The variation of the classification accuracy of the proposed Self-Organizing Fuzzy logic (SOF) classifier for the testing data was obtained by varying the granularity level (i.e., *L*) and the type of distance/dissimilarity measure. Furthermore, the variation of the execution time (i.e., *t*) of the classifier was also examined. The variation of the mean accuracy and the mean execution time with granularity level and distance type is given in Table 1. The mean accuracy was calculated by considering 10 different trained classifiers (10 randomly selected data set for training) for each case. The mean execution time was obtained by running the classifier 100 times for each case in a laptop with the Intel Core i7-9750H processor and 16 GB memory. It should be noted that the execution time represents only the time taken by the classifier when assigning the class of a given input, and it does not include the time taken by the vision processing module for extracting the inputs for the classifier.

Improvement in the accuracy with increasing *L* could be observed when the cosine distance was used. In contrast, a reduction in accuracy could be observed when *L* changed from 8 to 12 when Euclidean distance was used. The overfitting of the classifier was the reason for this behavior since *L* controls the generalization of the classifier. An increase in execution time could be observed with increasing *L* irrespective of the distance type. Nevertheless, the execution times are in the order of microseconds (highest 23.8 ms), implying a trivial computational overhead for the realtime operation of the robot.

The highest mean accuracy of 0.9942 was observed when Euclidean distance and granularity level of 8 were considered for the SOF classifier. The mean execution time for this configuration was 8.67 µs. Therefore, a trained case of Euclidean distance and granularity level of 8 was considered for the real-time experimenting with the robotic system. The confusion matrix corresponding to this trained SOF classifier is given in Table 2 for conveying insights of the classification performance.

### 4.2. Realtime Operation on the Robot

**Figure 9:**
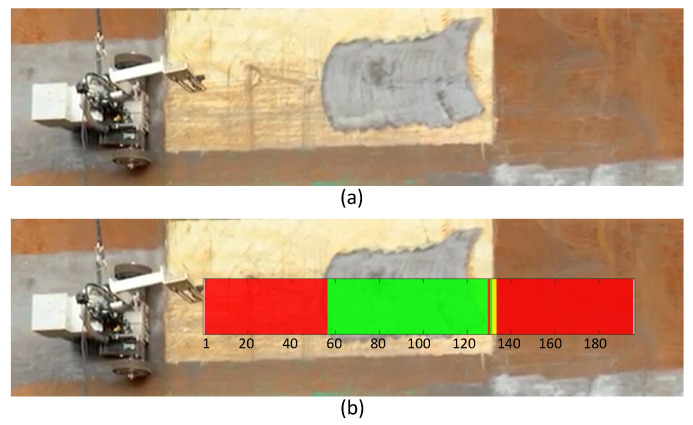
(**a**) Experimental setup; (**b**) The resulted benchmarking map. The benchmarking map is overlaid on the hull surface for better comparison. The areas predicted with good blasting quality by the system are given in green. The medium and bad quality areas are given in yellow and red, respectively. The corresponding frame number is annotated below the synthesized benchmarking map.

The proposed overall system has been implemented, and a benchmarking map generation has been tested on a ship hull. The benchmarking robot was placed in a ship hull, as shown in Figure 9. The ship hull had been blasted by the blasting robot previous to this experiment. The blasting robot would work usually covering the ship hull with more or less even blasting quality in most situations. However, areas with different blasting qualities could be observed due to the operational issues of the robot, such as interruptions of pressurized water supply and sudden variations in navigation speed, during the real operation for a long time. For the sake of better demonstration of the benchmarking ability, such interruptions had been intentionally created during the blasting. Intentionally interrupting the blasting robot for making different blasting performances would not detract the experimental condition from the spontaneity of typical operating conditions. After placing the benchmarking robot on the ship hull, the robot was moved in a horizontal path. The SOF classifier analyzed the visual feedback of the robot in realtime. A video (Supplementary Materials: Video S1) of this segment of the experiment is attached as a supplementary multimedia attachment.

**Figure 10:**
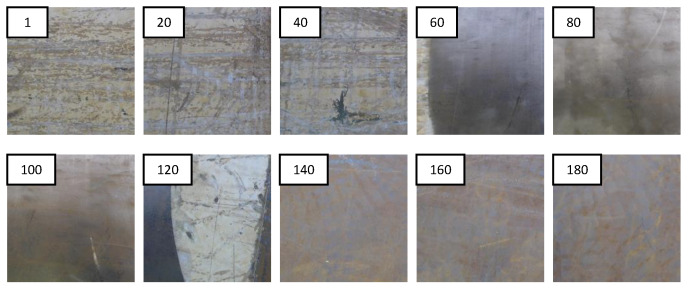
The image frames captured during the robot’s run with an interval of 20 frames. The corresponding frame number annotated in each image.

The robot could achieve a frame rate of 14.66 frames per second (fps). However, the frame rate was intentionally limited to 7 fps to avoid unnecessarily higher overlapping of the areas inspected by the robot in each frame. The corresponding benchmarking map generated by the system is overlaid on top of the ship hull, as shown in Figure 10. Here, the areas in green represent the good blasting quality, while the areas in yellow and red represent the areas with medium and bad blasting qualities. Altogether 195 captured frames were analyzed by the robot during this run, and the corresponding frame number is annotated in this overlaid map. The captured frames from the robot’s camera in intervals of 20 frames are given in Fig as samples for conveying the corresponding appearance and condition of the surface.

The area represented from frame 1 can be benchmarked as bad quality blasting based on expert knowledge. The corresponding color of the benchmarking map for the area represented by this frame is red, which indicates that the benchmarking method correctly identifies this frame. Similarly, the frames 20 and 40 were benchmarked as bad by the system as expected. The frames 60, 80, and 100 were benchmarked as good by the system as similar to human expert knowledge. However, frame 120 was benchmarked as good by the system, where the frame should not have been tagged as good per the human expert. The possible cause for this miss classification is that this image represents a combination of a good segment and a bad segment. The frames 140, 160, and 180 were tagged as bad in the benchmark map similar to the expert recommendation. Overall, the benchmarking robot was capable of correctly benchmarking the blasting quality to a greater extent. Nevertheless, few failure situations could be observed when the captured frames involve segments of different blasting qualities. Based on these observations, it can be concluded that the proposed benchmarking robotic system is capable of benchmarking the hydro blasting with adequate accuracy in realtime. Furthermore, it is capable of synthesizing a benchmarking map for a previously conducted hydro blasting.

The benchmarking map generated by the robot can be used to ensure the quality of a blasted ship hull before starting the painting. If the paint were applied to a ship hull that was not adequately blasted, the new paint would not last long, which degrades the quality of the maintenance work. Thereby, the proposed method for synthesizing a benchmarking map for hydro blasting would be useful to identify the areas that were not adequately blasted, and subsequently, selective spot blasting could be carried out on those areas. The benchmarking map would be useful in planning an efficient path for the blasting robot for the reblasting process in such cases. Furthermore, the representation of blasting quality categories in the map could be used to decide the amount of pressure required for the reblasting of the corresponding area. For example, if the benchmarking category is medium, a medium level of pressure can be used. In contrast, if the benchmarking category of an area is bad, then a high level of pressure can be applied for the corresponding area. Moreover, the categorical representation of the blasting quality in the benchmarking map would help to improve the overall efficiency of the blasting work. Therefore, the ability to synthesize the benchmarking map for hydro blasting work on a ship hull is widely useful for improving the efficiency and quality of ship hull maintenance.

A few miss classification was observed during the realtime operation with the robotic system. However, the accuracy of the proposed classifier is 99%, which is highly adequate for the application context. In addition to miss classification errors, the navigation errors of the robot due to poor localization cause errors to a synthesizing of a benchmarking map. Moreover, the accuracy of navigation and localization of the benchmarking robot should be maintained at a higher level to minimize the induce of errors. Nevertheless, solely relying on the beacon-based localization system is challenging in the working environment due to the high-frequency noises generated from the surrounding heavy-duty machinery. Therefore, the position estimations from the beacon system, inertial measurement unit, and wheel encoders are fused using the Kalman filter to improve the accuracy of the localization to an acceptable level.

The scope of this paper is limited to the design and development of a robotic benchmarking system for a robot-aided ship hull hydro blasting process. The proposed system is capable of synthesizing a benchmarking map for a previously blasted ship hull. Therefore, the work presented in this paper could contribute to improving the dry dock maintenance industry. Investigations on the development of methods for efficient path planning for a blasting robot and determination of optimum pressure for a selective blasting based on the information of a synthesized benchmarking map are proposed for future work.

In the current practice, the blasting quality is inspected by human experts for the decision making process. The blasting quality is determined based on qualitative factors of a blasted surface. This paper proposed a method to automate the inspection process. Nevertheless, the expert knowledge has to be captured for labeling the data set since a labeled data set is required for the training of the proposed classifier. Manual labeling of a large data set is a time-consuming task and would compromise the accuracy of the classifier if the expert knowledge was not captured accurately. During the labeling process, two human experts were asked to label the images independently. The label of an image is determined based on the unanimous decision of the human experts. Thus, this strategy ensures the correctness of the captured expert knowledge through manual labeling of the data set. The usage of unsupervised learning methods for the classification would alleviate the requirement of a labeled data set. The investigation on the possibility of using unsupervised learning methods for the classification is proposed for future work.

## 5. Conclusions

Routine dry dock maintenance work on ship hulls is essential for the efficient and sustainable operation of the shipping industry. In this regard, hydro blasting is one of the major maintenance work on ship hulls. Robots and systems have been developed for the dry dock maintenance industry to overcome the shortcoming of conventional methods.

This paper proposed a novel robotic system for benchmarking the robot-aided hydro blasting on ship hulls. The proposed robotic system is capable of synthesizing a benchmarking map that indicates the quality of a previously conducted hydro blasting in area wise. A novel Self-Organizing Fuzzy logic (SOF) classifier has been developed to realize the classification of blasting quality similar to human expert knowledge. A multipurpose inspection and maintenance robot called Hornbill, which can perform hydro blasting, benchmarking, and painting, has also been developed to facilitate a fully integrated system.

The SOF classifier has been trained and tested with a set of image data collected from the robot’s camera and labeled based on expert knowledge. The variation of the classification accuracy and the execution time of the SOF classifier with granularity level and distance/dissimilarity measure type has been studied to evaluate the classification performance. The highest achieved mean classification accuracy was 0.9942. The mean execution time of the classifier in the corresponding case was 8.67 µs, which indicates a trivial computational overhead to the system.

Real-time experimenting with the proposed robotic system has been conducted on a ship hull by navigating the benchmarking robot on a given path. According to the results, the proposed robotic system is capable of synthesizing a benchmarking map for a previously conducted hydro blasting process. A synthesized benchmarking map can represent the blasting quality of different areas in multilevel as similar to human expert categorization.

This benchmarking map can be used for planning an efficient navigation path for a blasting robot (for reblasting if a surface was not properly blasted) and by facilitating the spot blasting with different parameter settings based on identified blasting quality of the corresponding area. In addition to that, it can be used to verify whether the area of interest is adequality blasted before starting the painting. Therefore, the proposed robotic system would be highly beneficial in improving the quality and efficiency of hydro blasting work on the ship hull maintenance industry. Developments of methods for optimizing a reblasting process (robot-aided) based on a synthesized benchmarking map are proposed for future work.

## Figures and Tables

**Table 1:** Performance of the SOF classifier in different configurations.

Distance Measure	Euclidean			Cosine		
*L*	4	8	12	4	8	12
Accuracy	0.9744	0.9942	0.9928	0.9779	0.9915	0.9933
*t* (µs)	2.86	8.67	23.8	6.56	10.31	11.04

**Table 2:** Confusion matrix.

		Actual		
		Good	Medium	Bad
Predicted	Good	370	4	0
	Medium	0	366	1
	Bad	0	0	369

## Supplementary Materials

The following are available online at https://www.mdpi.com/1424-8220/20/11/3215/s1, Video S1: sensors-20-03215 supplementary.v3.
